# Effects of a Gluten-Containing Meal on Gastric Emptying and Gallbladder Contraction

**DOI:** 10.3390/nu10070910

**Published:** 2018-07-16

**Authors:** Sara Massironi, Federica Branchi, Mirella Fraquelli, Alessandra Baccarin, Francesco Somalvico, Francesca Ferretti, Dario Conte, Luca Elli

**Affiliations:** 1 Gastroenterology and Endoscopy Unit, Fondazione IRCCS Ca’ Granda Ospedale Maggiore Policlinico, 20122 Milan, Italy; federica.branchi@gmail.com (F.B.); mfraquelli@yahoo.it (M.F.); alessandra85.baccarin@gmail.com (A.B.); francesca.ferretti01@gmail.com (F.F.); dario.conte@unimi.it (D.C.); luca.elli@policlinico.mi.it (L.E.); 2 Department of Pathophysiology and Transplantation, Università degli Studi di Milano, 20122 Milan, Italy; 3 Alphasearch, 20100 Milan, Italy; alphasearch@tin.it; 4 Center for the Prevention and Diagnosis of Celiac Disease, Fondazione IRCCS Ca’ Granda Ospedale Maggiore Policlinico, 20122 Milan, Italy

**Keywords:** gluten, gastric emptying, cholecyst, celiac disease, non celiac gluten sensitivity

## Abstract

The ingestion of gluten has been associated with gastrointestinal symptoms even in the absence of detectable immune responses. Little is known about the pathophysiological effects of gluten on the upper gastrointestinal tract. We aimed to assess whether the ingestion of gluten leads to an impairment of the physiological mechanisms of gastric emptying, gallbladder contraction and relaxation. A total of 17 healthy subjects underwent ultrasound evaluation of gastric emptying dynamics and gallbladder contractions at baseline and every 30 min after a standard gluten-containing and gluten-free meal (250 kcal, 70% carbohydrates). The pattern of gastric emptying was similar after a standard meal with or without gluten, but differed in terms of the peak of the antral filling curve, which was wider (mean area 5.69, median 4.70, range 3.71‒9.27 cm^2^ vs. mean 4.89, median 4.57, 2.27‒10.22 cm^2^, *p* = 0.023) after the gluten-containing meal. The pattern of gallbladder contractions was different after the gluten-free meal (*p* < 0.05), with higher gallbladder volumes in the late refilling phases. The results of this study show that gluten ingestion exerts objective effects on gastric and gallbladder motility. Although the underlying pathophysiological mechanism remains unknown, these results could account for some of the gluten-related symptoms reported by patients with celiac disease and non-celiac gluten sensitivity.

## 1. Introduction

The ingestion of gluten-containing food has reportedly been involved in the development of a broad range of symptoms. Gluten is a structural protein found in wheat, barley and rye, composed of two main fractions, gliadins and glutenins [1]. In celiac disease (CD), the dietary intake of gluten leads to the intestinal exposure to gluten-derived peptides. This exposure triggers a T-cell mediated autoimmune reaction eventually leading to villous atrophy and malabsorption [2]. In wheat allergy (WA), an adverse IgE-mediated immune reaction develops against proteins contained in wheat (i.e., alpha-amylase/trypsin inhibitor, lipid transfer protein, gliadins, glutenins) with different clinical presentations [3]. However, recent data have suggested that the consumption of gluten-containing food can cause gastrointestinal symptoms even in the absence of detectable immune responses or signs of inflammation [4,5,6], which prompted the definition of a new class of gluten-related disorders, the non-celiac sensitivity (NCGS) or non-celiac wheat sensitivity. NCGS describes a spectrum of gastrointestinal and extra-intestinal symptoms occurring shortly after the dietary exposure to gluten and resolving when a gluten-free diet (GFD) has started [7,8,9]. Interestingly, GFD has shown benefits in terms of reduction of gastrointestinal symptoms when tested in a population with already known gastrointestinal disorders, such as inflammatory bowel disease or irritable bowel syndrome [10,11]. In spite of the growing evidence on the potential direct effects of gluten or related wheat-derived proteins on the gastrointestinal and immune system [12,13,14,15], there is still little data concerning the pathophysiological effects of gluten on the upper gastrointestinal tract. It is known that patients with CD tend to have higher fasting gallbladder volumes and slower gastric emptying before starting on GFD, possibly because of impaired levels of somatostatin and cholecystokinin [16,17]. However, these alterations of the upper gastrointestinal dynamics were considered more as a consequence of small bowel mucosal damage than a direct effect of gluten on the gastrointestinal system mediated by innate or adaptive immune response.

One can conjecture that, in subjects with disorders belonging to the spectrum of functional gastrointestinal disorders, as well as in those with a diagnosis of NCGS, the ingestion of gluten exerts a direct effect on gastrointestinal dynamics: this would explain the onset of symptoms following gluten-containing meals. Recent data have suggested that gluten may trigger a rapid innate immune response after ingestion also in subjects not affected by CD [15]. Magnetic resonance studies have showed that the ingestion of wholemeal bread results in delayed gastric emptying and reduced postprandial small bowel waters compared to a rice meal [18]. However, no significant effect on gastric emptying and small bowel and colonic distension was observed in a subsequent study investigating the effect of bread with different ingredients, including different gluten contents [19]. Ultrasound is a non-invasive, readily available technique that can be successfully applied in the setting of gastrointestinal dynamics [17,20]. To date, only a small-scale study has investigated, with the aid of ultrasound, the effect of gluten intake on healthy volunteers: it has shown no differences in gastric and gallbladder emptying in healthy volunteers after a gluten-containing vs. gluten-free meal [21]. With these premises, the aim of this study was to evaluate the effect of a gluten-containing meal on gastric emptying and gallbladder (GB) contraction and relaxation, as compared to a gluten-free isocaloric meal, in order to collect more data on the relationship between dietary intake of gluten and gastrointestinal dynamics.

## 2. Materials and Methods

### 2.1. Study Population

Between June 2014 and June 2015, 17 healthy volunteers were enrolled at the academic Gastroenterology and Endoscopy Unit, Fondazione IRCCS Ca’ Granda, Ospedale Maggiore Policlinico, Milan (Italy). The criteria for excluding the enrolment as healthy volunteers were: the presence of clinically relevant diseases (especially as concerns CD, food allergies and gastrointestinal diseases including functional gastrointestinal disorders), on-going pregnancy, active smoking habits. The absence of CD was proven by means of serological testing of tissue transglutaminase antibodies (TTG). All the subjects gave their written informed consent to the study, which was approved by the local ethics committee (n. 1491/2014). Basal clinical history data were collected and a physical examination was carried out for every subject. Anthropometric parameters (weight, height) were collected and the body mass index (BMI) was computed for each individual, using the standard formula: weight (kg)/height^2^ (m^2^).

### 2.2. Ultrasound Study of Gastric and Gallbladder Dynamics

The primary outcomes of the study were the assessment of changes in the gastric antrum size, in the dynamics of gastric emptying and of gallbladder contraction. All the subjects underwent the ultrasound evaluation of gastric and gallbladder dynamics twice: after a standard normocaloric balanced gluten-free meal and an isocaloric gluten-containing meal. Each subject received each meal in fasting conditions and after at least 72 h on a normocaloric balanced gluten-free diet.

### 2.3. Meal Administration

The composition of the administered meals was as follows:

- gluten-free meal—a standard fatty meal consisting of cooked egg albumen (110 g), two slices of toasted gluten-free bread (rice, millet and quinoa flour basis) (50 g), strawberry jam (20 g), water (120 mL). The meal contained 250 kcal, of which 70% from carbohydrates.
- gluten-containing meal—an isocaloric meal differing only as regards the type of bread, which was gluten-containing (wheat flour basis).

### 2.4. Ultrasonographic Evaluation

Gastric emptying and gallbladder motility were evaluated by real-time ultrasound examination with a Philips iU22 system (Philips Ultrasound, Bothell, WA, USA), equipped with a multi-frequency convex transducer (C5-2, 5-2MHz). The gastric antral area was measured three times and the mean values of the two diameters were used to calculate the area, assuming an elliptical shape, by means of the formula: πAB/4, where A is the longitudinal diameter and B the anteroposterior diameter as measured at the cross-section of the gastric antrum, corresponding to the sagittal plane passing through the superior mesenteric vein, according to Bolondi et al. [20]. The basal antral measurements were taken after overnight fasting (basal time, 0 min). After having the standard meal, all the subjects underwent US measurements every 30 min until complete gastric emptying. Gastric emptying was considered ended when the antral area returned to basal values and remained then unchanged for at least 30 min. The gallbladder volume was calculated by the ellipsoid formula: πAxBxC/6 as previously described by Dodds et al. [22], where A is the longitudinal, B the transverse and C the antero-posterior diameter. Gallbladder measurements were taken at time 0 and every 30 min after the standard meal, until the refilling occurred after an initial contraction. Also for the gallbladder three different measurements were taken in rapid sequence and the mean values were used. The gallbladder residual volume (mL) was the smallest volume measured at the completion of the meal-induced emptying. The in-percent difference between the basal volume and the residual volume represented the gallbladder ejection volume (mL) (GB EF%). All the examinations were obtained and evaluated in real time by two expert operators (SM and AB) blinded as regards the type of meal received by the participants. At the end of the observation time, all subjects were asked to fill in a questionnaire regarding the onset of common dyspeptic symptoms after each meal, i.e., upper abdominal pain or discomfort, nausea, vomiting, fullness, bloating, early satiety and belching, as well as their intensity measured by means of visual analogic scales ranging from 0 to 10.

### 2.5. Statistical Analysis

This study was designed as a pilot study and a sample size estimation was not considered possible as this preliminary stage. Demographic and clinical data were expressed as medians and ranges or means and SD, unless stated otherwise. The difference in mean gastric antral area variations after the two meals was evaluated by means of ANOVA or Friedman’s model. The areas under the curve were calculated for both the gastric emptying and gallbladder contraction dynamics and the differences were analyzed with the paired *t*-test or the Wilcoxon matched-pairs signed rank test. The presence of any association between the onset of symptoms and variation in the gastric antral area or other US parameters was evaluated by means of Pearson’s correlation test. All the data were tested for a normal distribution using the Kolmogoroff–Smirnoff test. Statistical analysis was performed with the software package SPSS v. 19 (IBM SPSS Statistics, rel. 2010, Armonk, NY, USA: IBM Corp). A two-tailed *p* value < 0.05 was considered statistically significant. 

## 3. Results

The study population consisted of 17 subjects, 11 females and six males, with a median age of 30.5 years (range 25–41) and a median BMI of 20.8 Kg/m^2^ (range 17.7–29.9). No participant had relevant concomitant conditions nor drug intake, nor alcohol consumption (defined as more than 1 alcoholic unit per day). Only one participant was a smoker (five cigarettes a day).

**Figure 1:**
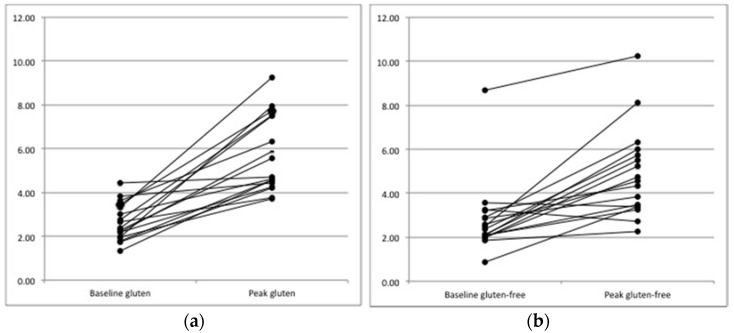
Changes in the gastric antral area at peak of the 17 healthy volunteers after gluten-containing (**a**) and gluten-free meals (**b**); *p* = 0.01. *y*-curve: area, cm^2^.

**Figure 2:**
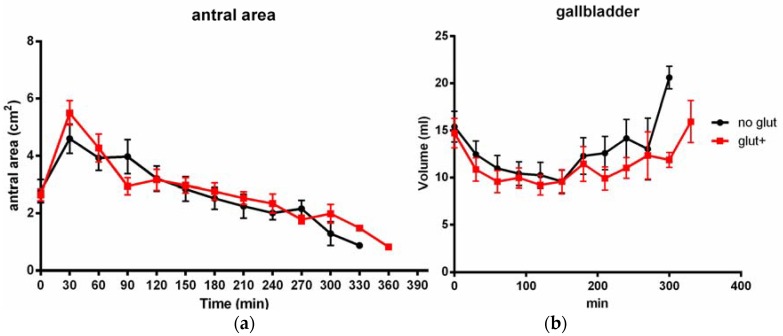
Detailed gastric filling and emptying patterns (**a**) and gallbladder contraction (**b**) after the two meals. Data are shown as mean (standard deviation).

After the gluten-containing meal, the postprandial filling peak was significantly wider than after the gluten-free meal (median antral area 4.70 cm^2^, range 3.71–9.27 vs. 4.57 cm^2^, range 2.27–10.22, *p* = 0.025, Figure 1). Also the mean difference between the basal antral area and the antral filling curve peak was significantly higher after the gluten-containing meal (3.02 ± 1.74 cm^2^ vs. 1.89 ± 1.68 cm^2^, *p* = 0.01). Overall, gastric emptying dynamics were similar for both types of meals (Figure 2) and the time-to-peak of the antral filling curve was not significantly different between the two meals (mean 45.9 ± 30.2 min after the gluten-containing meal vs. 52.9 ± 34.4 min after the gluten-free meal, *p* = 0.47). Regarding gastric emptying, the emptying time was slightly longer after the gluten-containing meal than after the gluten-free meal, although this finding was not statistically significant (mean 222.35 ± 81.5 min vs. 202 ± 64.97 min, *p* = 0.37). The results are summarized in Table 1. Age, sex and BMI did not correlate with the gastric filling curve nor did they with the time-to-peak and the gastric emptying time.

The gallbladder contraction patterns were different between the gluten-containing and the gluten-free meal aftermaths (area under the curve, AUC: 3.711 vs. 3.630, *p* = 0.001), with higher gallbladder volumes being observed especially in the late refilling phases (*p* = 0.039 at 240 min, <0.01 at 300 min). The gallbladder ejection fraction did not differ between the two meals. The fasting gallbladder volume did not correlate with age, sex, BMI nor with the basal antral area (Table 2). Interestingly, no subject reported the onset of gastrointestinal symptoms after either meal.

## 4. Discussion

The results of our study show that the ingestion of a gluten-containing meal may cause specific effects on the upper gastrointestinal dynamics irrespective of the presence of a gluten-related disorder. In the group of healthy volunteers we examined, small but significant differences were observed in both the gastric filling and gallbladder contraction dynamics. Regarding gastric filling and emptying dynamics, a significantly wider antral area was observed after a gluten-containing meal than after the isocaloric gluten-free meal, although the filling and emptying times and the general dynamic pattern were not altered by the different composition of meals. The gallbladder contraction after the two meals did not vary significantly as regards the entity of the contraction, but after the gluten-containing meal a longer contraction time was observed, with a significantly longer time required to return to the basal fasting volume.

In patients with known gastrointestinal disorders, the ingestion of gluten-containing food exerts upper gastrointestinal symptoms which are to date not commonly explained from a pathophysiological point of view (in the case of NCGS) or only partially explained by the immune response to gluten (in the case of celiac disease). Thus, this study was performed in order to objectively analyze the presence of differences in gastric and/or gallbladder motility according to the content of gluten in a meal, irrespective of the onset of symptoms on healthy subjects. The differences we observed, though not dramatic, appear to confirm the hypothesis that the ingestion of gluten may exert a direct effect on the gastrointestinal tract and thus cause symptoms through a pathogenetic mechanism other than those associated with the development of celiac disease.

In fact, gluten, or gluten-related peptides, can reportedly elicit several reactions when they are in contact with the gut and in particular with immune cells [15,23,24]. The alteration of gut permeability, as a consequence of a direct effect of gluten-derived peptides or through the triggering of an immediate innate immune response, has been studied in both animal models and human subjects with the aim of explaining the pathogenesis of both celiac disease and NCGS [23,24]. Moreover, the increase in the secretion of hormones, such as insulin, links to the exposure to gliadin peptides in animal models and in vitro [25]. A direct link between gluten and the secretion of gastrointestinal hormones has not been demonstrated at a molecular level; however, one can suppose that the interaction between gluten-derived peptides and immune cells in the intestinal mucosa and submucosa might also directly influence the secretion of these hormones and thus the dynamics of gastric emptying and gallbladder contractions [17,26]. This is in line with the observation of an impaired gallbladder motility in patients with celiac disease in relation to a reduced secretion of enteric hormones and/or decreased gallbladder sensitivity to them. In particular, untreated celiac patients, when compared to controls, have showed low levels of postprandial cholecystokinin and increased fasting somatostatin levels [17]. Moreover, a gastric emptying time longer than in controls has been observed in untreated celiac patients, along with the concomitant increase in plasma neurotensin levels [26].

Other mechanisms that possibly explain our observations, particularly the potential role of proteins other than gluten on the gastric and gallbladder motility are: the increased gastric filling observed at ultrasound after the gluten-containing meal which is possibly a consequence of other wheat components, such as amylase-tripsin inhibitors (ATI) [13] or fructans, which have been linked to the development of gastrointestinal symptoms in both patients with NCGS and irritable bowel syndrome [27,28,29]. However, this study is limited since the exact content of ATI and fructans in our meals was not calculated. Further data would be useful to directly compare the effect of ATI, fructans and gluten on the upper gastrointestinal dynamics.

As for the limitations of our study, first of all the small sample size may have led to less precision of the estimates; further studies will have to be designed in order to overcome this limit. Moreover, the group of participants was not entirely balanced as it included more women (11/17, 65%). However, considering the cross-over design of the study, every patient acted as their own control, which helps reduce biases. Finally, the investigation of gastric and gallbladder dynamics by means of ultrasound is not the standard methodology for the diagnosis of altered gastric emptying, although it is easy and non-invasive and has already been evaluated in previous studies [17,20,21,22].

It is worth noting that none of our healthy subjects reported the onset of gastrointestinal symptoms after the meals, irrespective of the actual changes on gastric volume or gallbladder dynamics observed. It is known that in subjects with functional gastrointestinal disorders, alterations in visceral motility or distension considered within “normal” range are associated with the onset of symptom [30]. One can hypothesize that in patients reporting symptoms after having a gluten-containing meal, the alterations caused by the meal would be perceived differently, thus corroborating the hypothesis of an enhanced visceral sensitivity in patients with the irritable bowel syndrome spectrum [30]. Of course, numerous other factors could impact symptoms and responses in patients, including innate immune reactions, size of the meals, so that the specific role of gluten still has to be clarified. In order to prove this speculative hypothesis, further studies should investigate the effects of meals with different gluten content on patients with IBS or NCGS.

## 5. Conclusions

In conclusion, the results of our study support the hypothesis of a direct effect of gluten intake on both gastric and gallbladder motility, especially regarding the dynamics of gastric filling and the gallbladder contraction pattern. Although the underlying pathophysiological mechanism is still unknown, these observations could, if substantiated by further studies on patients, explain some of the gluten-related symptoms reported by patients with NCGS and other gastrointestinal disorders, who are improving through GFD.

## Figures and Tables

**Table 1:** Parameters of the gastric dynamics after gluten-containing and gluten-free meals.

Parameters	Gluten-Containing Meal	Gluten-Free Meal	*p*
Gastric filling peak area in cm^2^, median (range)	4.70 (3.71–9.27)	4.57 (2.27–10.22)	0.023
Difference peak-basal area in cm^2^, median (range)	3.02 ± 1.74	1.89 ± 1.68	0.01
Time to peak mins, mean (SD)	45.9 ± 30.2	52.9 ± 34.4	0.47
Gastric emptying time mins, mean (SD)	222.4 (81.5)	202 (65.0)	0.37

SD: standard deviation.

**Table 2:** Parameters of the gallbladder dynamics after gluten-containing and gluten-free meals.

Parameters	Gluten-Containing Meal	Gluten-Free Meal	*p*
GB basal volume mL, mean (SD)	14.7 (6.5)	15.4 (6.8)	0.76
Gallbladder ejection fraction %, mean (SD)	44.4 (28.1)	44.2 (28.1)	0.9
GB volume at 240 min mL, median (range)	10.8 (5.2–13.4)	14.3 (12.1–21.2)	0.039
Pattern of GB contraction mL*min, AUC	3711	3630	0.001

GB: gallbladder; AUC: area under the curve.
